# The relationship between disability and quality of life among older adults: the roles of sleep, depression, and living arrangements

**DOI:** 10.3389/fpubh.2026.1854817

**Published:** 2026-07-01

**Authors:** Jing Zhu, Zhiqiang Feng, Zhongming Chen, Lin Guo, Chengxin Fan, Chunxiao Yang, Zixuan Zhao, Xingang Sang, Lingzhong Xu, Wenqiang Yin

**Affiliations:** 1 School of Public Health, Shandong Second Medical University, Weifang, Shandong, China; 2 “Health Shandong” Severe Social Risk Prevention and Management Synergy Innovation Center, Weifang, Shandong, China; 3 School of Management, Shandong Second Medical University, Weifang, Shandong, China; 4 Weifang Municipal Health Commission, Weifang, China; 5 Centre for Health Management and Policy Research, School of Public Health, Cheeloo College of Medicine, Shandong University, Jinan, Shandong, China; 6 NHC Key Laboratory of Health Economics and Policy Research, Shandong University, Jinan, China

**Keywords:** older adults, disability, depression, sleep quality, quality of life, living arrangement, public health

## Abstract

**Background:**

The acceleration of population aging, especially in China, has led to a rapid growth in the number of older adults living with disabilities, posing significant challenges to public health and quality of life (QoL). From the perspective of the biopsychosocial framework, the adverse impact of disability (a biological and functional factor) on QoL has been well documented. However, the underlying mechanisms linking disability to QoL within this framework, especially the mediating roles of psychological factors (e.g., depression) and physiological correlates (e.g., sleep quality), remain insufficiently understood. Furthermore, living arrangements, as a key social factor in the biopsychosocial model, may moderate these mediating pathways. Therefore, this study aimed to examine the mediating roles of sleep quality and depression in the association between disability and QoL and to explore whether living arrangements moderate these pathways within the biopsychosocial framework.

**Methods:**

A total of 3,855 adults aged ≥60 years were recruited through the Household Health Interview Survey in Taian City, China, using a multi-stage stratified cluster sampling method. Data were collected on disability [measured using the Physical Self-Maintenance Scale (PSMS)], sleep quality [assessed using the Pittsburgh Sleep Quality Index (PSQI)], depression [assessed using the Patient Health Questionnaire-9 (PHQ-9)], living arrangements, QoL (assessed using the EQ-5D-5L), and relevant covariates. Statistical analyses included descriptive analysis, logistic regression, and moderated mediation analysis using the PROCESS macro.

**Results:**

Disability was significantly associated with poorer QoL (OR = 1.816–1.928, *P* < 0.001), total effect B = 0.767). Sleep quality and depression partially mediated this association via both parallel and serial pathways, with the serial mediation effect through sleep quality and depression being significant (B = 0.046, *P* < 0.001). The total indirect effect accounted for 17.00% of the total effect. Furthermore, living arrangements significantly moderated the associations of disability with sleep quality and depression, with stronger adverse effects observed among older adults living alone.

**Conclusion:**

Disability was associated with poorer QoL among older adults, and this relationship was partially explained by sleep quality and depression, with living arrangements further moderating the pathways linking disability to sleep quality and depression. These findings highlight the importance of integrated interventions that address physical, psychological, and social factors to improve QoL among older adults with disabilities. Targeted strategies, including sleep management, depression screening, and enhanced social support, particularly for those living alone, may help improve QoL and promote healthy aging.

## Graphical abstract

**Figure:**
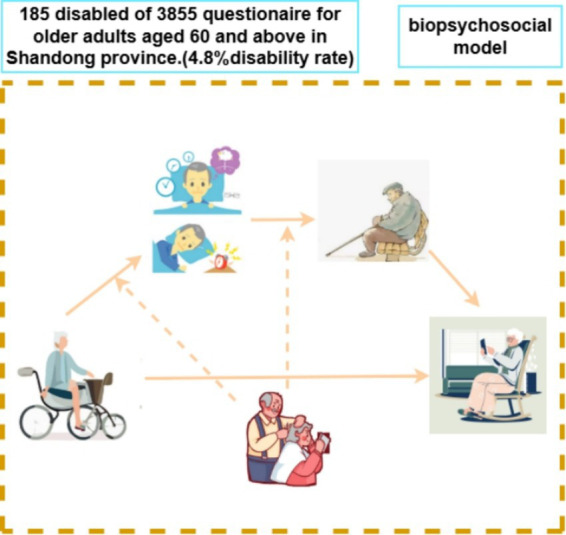

## 1 Introduction

Population aging is a major global public health challenge, with China experiencing a particularly rapid increase in its older population. By 2020, the proportion of the Chinese population aged 60 years and above had risen to 18.7% (264 million), an increase of 5.44 percentage points compared with 2010 (1). This trend is expected to intensify, with forecasts suggesting that the number of people aged 60 years and over will reach 400 million by 2035 (2). Of greater concern, the number of disabled and semi-disabled older adults in China has already exceeded 40 million and is projected to reach 58 million by 2050 (3, 4). This rapid growth is associated with an increasing burden of age-related chronic diseases and long-term care needs (5). Unprecedented global population aging poses substantial challenges to public health systems. More importantly, this trend has seriously undermined the health-related quality of life (QoL) and overall wellbeing of older individuals (6).

Against this backdrop, to enhance the QoL of older adults with disabilities, the World Health Organization (WHO) introduced the “active aging” policy framework in 2002, emphasizing the optimization of opportunities for health, participation, and security to improve QoL among older populations (7). In 2015, the WHO further expanded this framework to the concept of “healthy aging” (8). Consistent with these global initiatives, the present study explores the mechanisms influencing QoL among older adults with disabilities. For this study, disability is defined as the impairment of physical, cognitive, or emotional functions that limits older adults’ ability to perform activities of daily living (ADLs) (9). QoL is an important indicator of population health and wellbeing. It is a comprehensive concept that encompasses biological, psychological, and social adaptation, as well as happiness (10, 11). Existing studies have consistently shown that disability significantly reduces the QoL of older adults (12, 13). To clarify how disability is linked to QoL through multiple pathways, the present study adopts the biopsychosocial (BPS) model as an integrated analytical framework (6, 14). According to the BPS model, disability, as a biological factor, is linked to QoL not only through direct paths but also indirectly through psychological and social mechanisms. These potential pathways will be further explored in the following sections.

While the direct negative impact of disability on QoL has been well-established (15, 16), the underlying mechanisms linking disability to QoL remain insufficiently understood among older adults. Emerging evidence suggests that both biological and psychological pathways may play important roles in this relationship. Studies have revealed a link between disability and sleep quality, as well as between sleep quality and QoL (17, 18). From a biological perspective, disability is often accompanied by chronic conditions, pain, and functional limitations, which may impair sleep quality (19). Poor sleep, in turn, has been associated with cognitive decline, an increased risk of chronic diseases, and reduced overall health status, ultimately contributing to lower QoL among older adults (18, 20). From a psychological perspective, declines in functional capacity may be related to feelings of helplessness and reduced social engagement, which in turn increase the risk of depression. Previous studies have consistently identified these psychosocial pathways as key mechanisms linking functional limitations to mental health outcomes (21, 22). An association also exists between depression and QoL (23). Furthermore, sleep disturbances often present before the onset or recurrence of a severe depressive episode. This temporal relationship has been verified in earlier investigations (24). Moreover, this finding suggests a close interplay between sleep and mental health. To summarize, these findings indicate a potential serial mediation pathway in which disability may impair sleep quality, which subsequently exacerbates depressive symptoms, ultimately leading to lower QoL. However, few studies have examined these interrelated mechanisms simultaneously, particularly among older adults. Therefore, it is necessary to further investigate the mediating roles of sleep quality and depression in the association between disability and QoL.

Furthermore, the association between disability and QoL may be linked to social factors, particularly living arrangements among older adults. Living arrangements, as an important indicator of social support, can affect physical and psychological wellbeing (25). According to the social support stress-buffering model proposed by Cohen et al., social support can mitigate the negative effects of stressors by providing emotional and instrumental resources (26, 27). In this context, co-residing with family members may help buffer the adverse consequences of disability by providing daily care and emotional support. In contrast, older adults living alone may experience reduced access to social and material resources. The Conservation of Resources (COR) theory suggests that resource loss or insufficient resource gain can increase vulnerability to stress, thereby negatively affecting health outcomes (28, 29). Thus, living arrangements may serve as a moderator in the association between disability and its subsequent outcomes, such as sleep quality and depression, which are further linked to QoL.

Despite growing evidence on the associations between disability, sleep quality, depression, living arrangements, and quality of life (QoL), the underlying mechanisms linking these factors remain insufficiently understood. In particular, few studies have simultaneously examined the potential mediating roles of sleep quality and depression in the relationship between disability and QoL, as well as the moderating role of living arrangements within this framework. Guided by the biopsychosocial model, this study aims to investigate a moderated mediation model that integrates mediating mechanisms and moderating effects linking disability to QoL among older adults. The following hypotheses are proposed:

*H1*: Sleep quality and depression mediate the association between disability and QoL among older adults.

**Figure 1:**
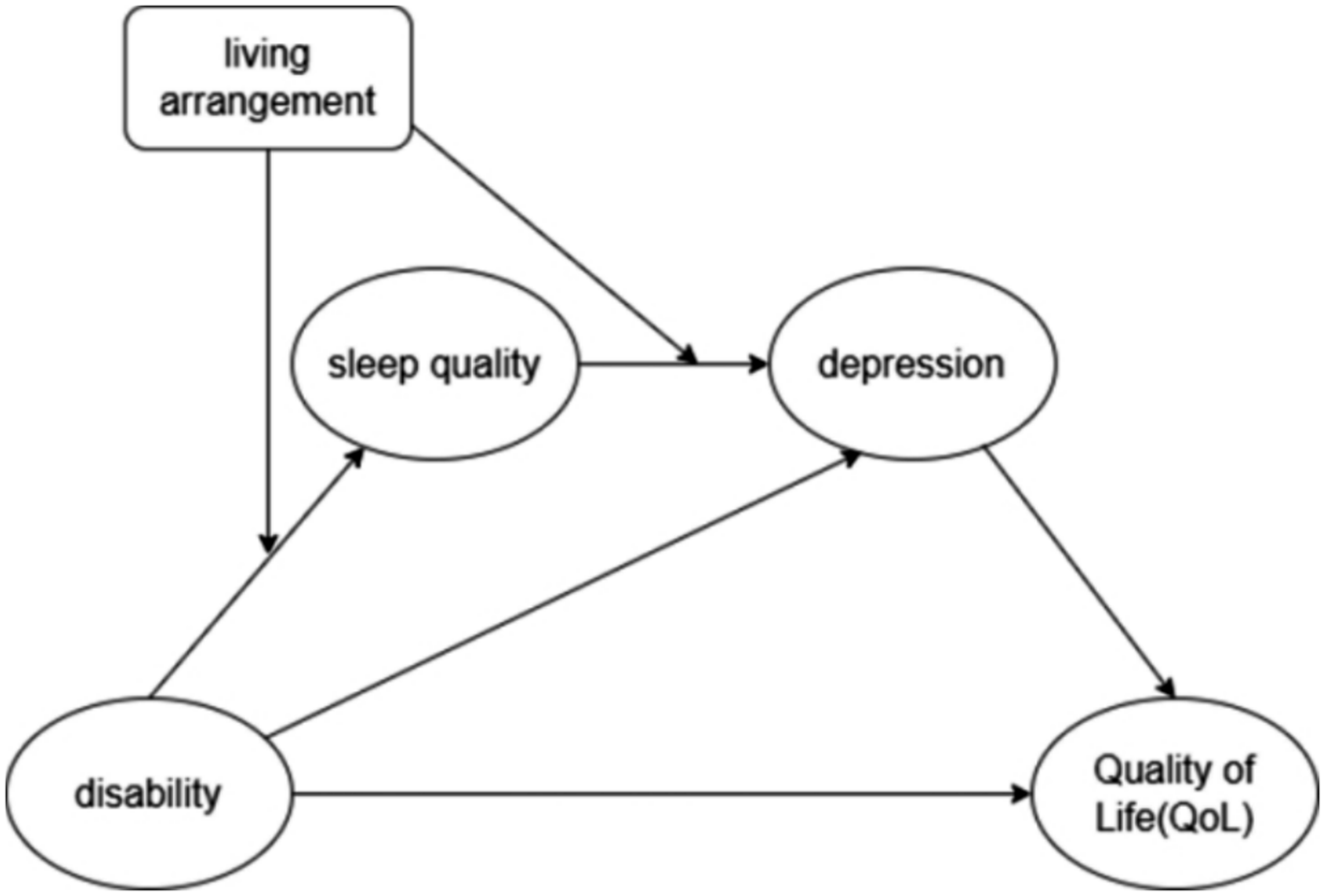
The theoretical hypothetical model.

*H2*: Living arrangements play a moderating role in the association of disability with sleep quality and depression (Figure 1).

In the context of rapid global population aging and a growing number of older adults living with disabilities, this issue carries significant public health implications. From this perspective, identifying the key factors that are linked to QoL, as well as the underlying mechanisms, is essential. Such efforts can support policymakers in developing targeted interventions that aim to reduce risk factors and strengthen protective factors. Finally, addressing these interconnected factors is critical for promoting healthy and active aging.

## 2 Methods

### 2.1 Study design and participants

**Figure 2:**
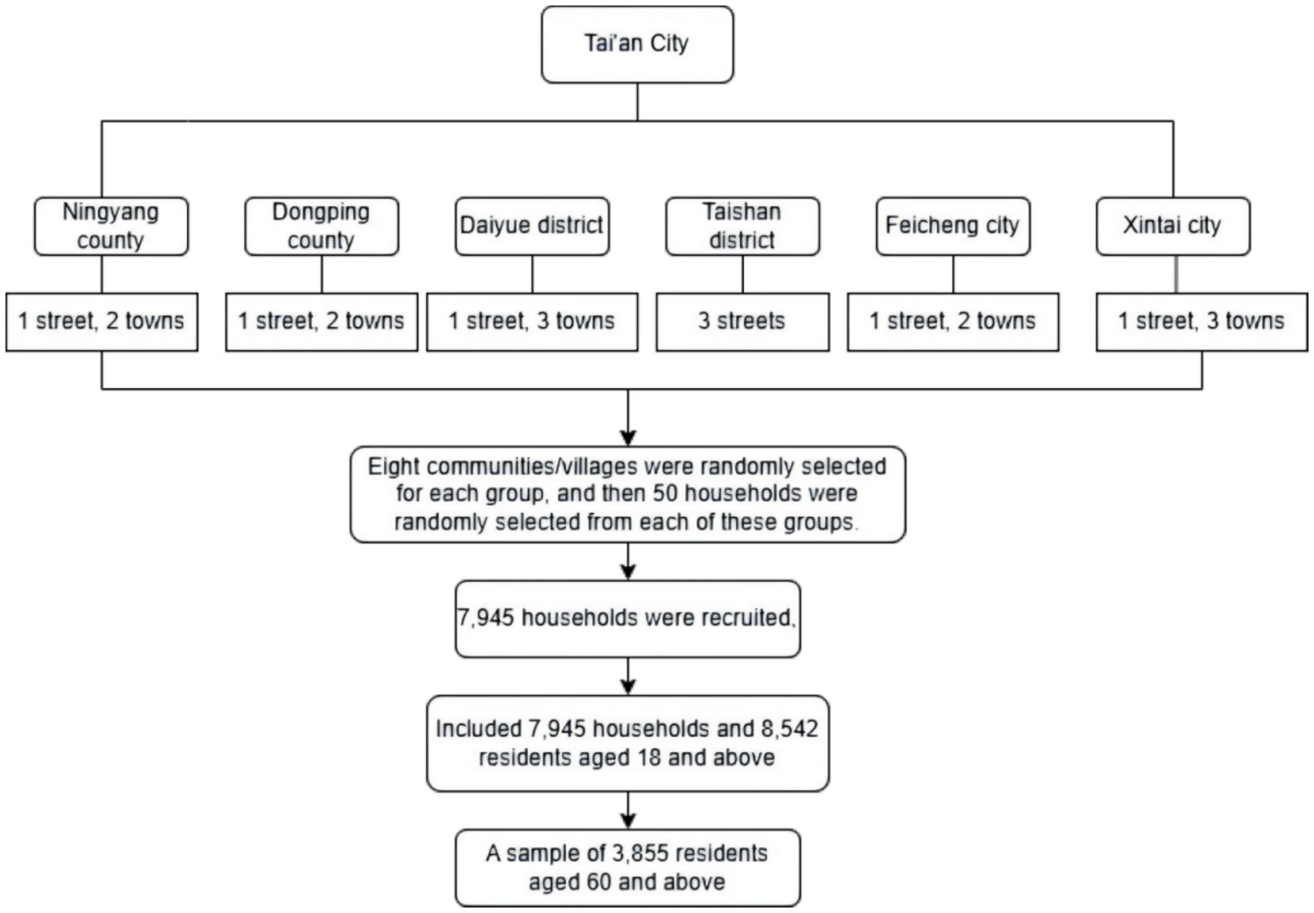
Sampling flow chart.

Considering the need to investigate population health status and health service demand, this research was designed as a cross-sectional study and conducted in Taian City, Shandong Province, where population aging and the demand for care services for older adults with disabilities have become increasingly prominent (30, 31). These data were collected via a large-scale population survey and were used to examine residents’ health status and their demand for and utilization of health services. To ensure representativeness, stratified multi-stage random sampling was employed throughout the survey process. Participants were selected from all six administrative districts of the city, including four counties and two districts. The sampling strategy was consistent with that used in our previous study to maintain comparability across research findings (32). First, in the initial stage, sub-districts and towns were stratified by high, medium, and low socioeconomic development levels and categorized by geographical location into urban, suburban, and rural areas, and then sampled accordingly. The probability proportionate to size (PPS) sampling method was applied. Using this approach, three sub-districts or towns were randomly chosen from each county or district in Taian City. Second, within each selected town and sub-district, villages and committees were sampled. Eight villages and eight committees were selected separately using PPS. In total, 160 villages and committees were included. Finally, households were selected within each village or committee. On average, 50 households were randomly chosen using simple random sampling. These households constituted the final sample. Eligible participants were individuals aged 18 years or older with local household registration who could clearly understand and express their opinions. A total of 7,945 households were recruited. Ultimately, the final analytical sample consisted of 7,920 households with a total of 8,542 participants, with an effective response rate of 99.7%. For this study, only older adults aged 60 years and above were included. Accordingly, 3,855 older adults were selected for analysis. The detailed participant screening and sampling procedure is presented in Figure 2. Before data analysis, missing values were screened and properly handled to ensure data quality. All participants were interviewed face-to-face by trained interviewers in the participant’s home using a paper-based questionnaire. The study protocol was approved by the Ethical Committee of the Center for Health Management and Policy Research, Shandong University (approval number: LL20191220). The participants provided their written informed consent to participate in this study.

### 2.2 Measures

QoL: QoL assessment in the study was conducted using the EQ-5D-5L scale. The scale consisted of five dimensions: mobility, self-care, daily activities, pain/discomfort, and depression/anxiety. Each dimension had five levels of responses: no difficulty, slight difficulty, moderate difficulty, severe difficulty, and extremely severe difficulty. The EQ-5D-5L has been widely validated and has demonstrated good reliability and applicability among Chinese community-dwelling older adults in previous studies (33, 34). This study converted the health conditions of the subjects in the five dimensions into health utility values based on the EQ-5D-5L value set released by Luo et al. in 2017 (35). The range of health utility values is from −0.391 (with extreme difficulty in all five dimensions) to 1.000 (with no difficulty in all five dimensions). For analytical purposes, to reduce the ceiling effect and based on previous studies, the lower limit of the interquartile range was used as the standard (36, 37). The utility values were catagorized into “Good” (≥0.8616) = 0 and “Poor” (<0.8616) = 1 groups. The Cronbach’s *α* coefficient of this scale was 0.798, indicating good internal consistency and reliable measurement results. Additionally, this dichotomous classification is more intuitive for clinical and public health practice, facilitating the clear identification of high-risk groups that need targeted intervention. It also effectively avoids the ambiguity caused by continuous variable interpretation, ensuring the rigor and rationality of the research results.

Disability: In this study, the assessment of the disability status of the older adults was conducted using the Physical Self-Maintenance Scale (PSMS) from the Activities of Daily Living (ADL) scale (38). This scale consists of six items: dressing, eating, bathing, getting in and out of bed, using the toilet, and continence (urination and defecation control). Each item was scored on four levels (1–4): no difficulty, having difficulty but still able to complete independently, having difficulty and needing help, and being unable to complete. The total score is 24 points. A score of >6 indicates disability. Higher scores indicate more severe disability. The Cronbach’s *α* coefficient for this scale was 0.964.

Sleep quality was assessed using the Pittsburgh Sleep Quality Index (PSQI) (39). The scale consists of 19 items. These items are grouped into seven dimensions: subjective sleep quality, sleep latency, sleep duration, habitual sleep efficiency, sleep disturbances, use of hypnotic drugs, and daytime dysfunction. Each dimension is scored from 0 to 3 based on the corresponding items. The total score ranges from 0 to 21. Higher scores indicate more severe sleep problems. This scale has been widely used in numerous studies and has demonstrated high reliability and validity (40). In the present study, the Cronbach’s *α* coefficient of the PSQI was 0.757. The Kaiser–Meyer–Olkin (KMO) measure of sampling adequacy was 0.741, and Bartlett’s test of sphericity was statistically significant (*p* < 0.001). These results indicate good reliability and validity of the PSQI.

Depression among older adults was assessed using the Patient Health Questionnaire-9 (PHQ-9) (41). The scale includes nine items. These items reflect individuals’ experiences over the past 2 weeks. Responses are rated on a 4-point frequency scale, ranging from 0 (did not apply to me at all) to 3 (applied to me very much). The total score ranges from 0 to 27. Higher scores indicate more severe depressive symptoms. The PHQ-9 is a valid and reliable measure of depression severity (42–44). In the present study, the Cronbach’s *α* coefficient for the PHQ-9 was 0.861.

Living arrangements: In this study, we used the question “How many people were living in your household in the past 6 months?” to evaluate the living conditions of the older adults, combined with inquiries about the type of cohabitants (focusing on family members such as spouses and children, consistent with the household survey setting). The responses of the respondents were categorized as living alone (1 point) or not living alone (0 points). It was used in a previous study (45), which validated its rationality in geriatric household survey research, ensuring the consistency and reliability of the research design. Specifically, “living alone” refers to older adults who live in a household without any cohabitants, while “not living alone” refers to older adults who live with family members (such as spouses, children, or other relatives) in the same household.

Covariates: Based on univariate analysis and previous literature, the following covariates that may be related to QoL were included: sex, age, education level, health insurance type, household income, smoking status, alcohol consumption, exercise frequency, and the presence of chronic diseases (46, 47).

### 2.3 Statistical analysis

Data analysis was performed using SPSS 26.0 (IBM Corp., Armonk, NY, USA) software package. First, descriptive statistics and univariate analyses (chi-square tests for categorical variables and *t*-tests for continuous variables) were conducted to examine the differences in characteristics between the “Good” and “Poor” QoL groups (Table 1).

**Table 1:** Characteristics and univariate analysis of quality of life among older adults.

Characteristics	Total	QoL		*t/χ^2^*
	3,855	≥0.8616	<0.8616	
Sex [*n* (%)]				19.916^***^
Male	1,562 (40.5)	1,318 (84.4)	244 (15.6)	
Female	2,293 (59.5)	1803 (78.6)	490 (21.4)	
Age (years), mean ± SD	68.910 ± 5.979	68.620 ± 5.888	70.061 ± 6.222	−5.900^***^
Degree of education [*n* (%)]				63.637^***^
Illiterate	1720 (44.6)	1,308 (76.0)	412 (24.0)	
Primary school	823 (21.3)	667 (81.0)	156 (19.0)	
Junior school	833 (21.6)	718 (86.2)	115 (13.8)	
High school or above	479 (12.4)	428 (89.4)	51 (10.6)	
Health insurance [*n* (%)]				23.874^***^
Medical insurance for urban employees	460 (11.9)	411 (89.3)	49 (10.7)	
Urban and rural residents’ medical insurance	3,348 (86.8)	2,672 (79.8)	676 (20.2)	
Others	47 (1.2)	38 (80.9)	9 (19.1)	
Household income [*n* (%)]				48.189^***^
Quarter 1	975 (25.3)	732 (75.1)	243 (24.9)	
Quarter 2	1,052 (27.3)	832 (79.1)	220 (20.9)	
Quarter 3	991 (25.7)	830 (83.8)	161 (16.2)	
Quarter 4	837 (21.7)	727 (86.9)	110 (13.1)	
Smoking status [*n* (%)]				8.805^***^
Current smoker	583 (15.1)	493 (84.6)	90 (15.4)	
Former smoker	471 (12.2)	392 (83.2)	79 (16.8)	
Never smoked	2,801 (72.7)	2,236 (79.8)	565 (20.2)	
Alcohol consumption [*n* (%)]				22.842^***^
Almost daily	643 (16.6)	560 (87.1)	83 (12.9)	
Frequently	207 (5.4)	170 (82.1)	37 (17.9)	
Occasionally	308 (8)	243 (78.9)	65 (21.1)	
Former drinker	2,697 (70)	2,148 (79.6)	549 (20.4)	
Exercise [*n* (%)]				84.489^***^
Never	1,487 (38.6)	1,101 (74.0)	386 (26.0)	
1–2 times/week	149 (3.9)	117 (78.5)	32 (21.5)	
3–5 times/week	219 (5.7)	176 (80.4)	43 (19.6)	
≥6 times/week	2000 (51.9)	1727 (86.4)	273 (13.7)	
Chronic disease [*n* (%)]				139.066^***^
Yes	2,851 (74)	2,182 (76.5)	669 (23.5)	
No	1,004 (26)	939 (93.5)	65 (6.5)	
Disability score, mean ± SD	6.217 ± 1.217	6.035 ± 4.429	6.989 ± 2.493	−20.077^***^
Sleep quality, mean ± SD	5.680 ± 4.503	5.029 ± 4.098	8.428 ± 5.098	−19.191^***^
Depression, mean ± SD	3.046 ± 3.834	2.295 ± 2.887	6.240 ± 5.402	−27.418^***^

SD, standard deviation; QoL, quality of life; *t*, *t*-test; *χ*^2^, chi-square test; ^***^p < 0.001.

Based on the theoretical framework of the bio-psycho-social model and the hypothesized pathways among disability, sleep quality, depression, living arrangements, and QoL, the mediation model and moderated mediation model were appropriately adopted to examine the sequential mediating roles of sleep quality and depression, as well as the moderating effect of living arrangements. Subsequently, regression analyses were performed to examine the associations among disability, sleep quality, depression, living arrangements, and QoL (Table 2). In the binary logistic regression, Model 1 included disability and all covariates. Model 2 additionally included sleep quality (*M*1), and Model 3 further included depression (*M*2) to examine changes in the association between disability and QoL before formal mediation testing. Models 4 and 5, using linear regression, specifically tested the interaction effect between disability and living arrangements on sleep quality and depression, respectively. Finally, the SPSS macro program PROCESS V4.0 was used to verify the moderated mediation model. Specifically, Model 84 was applied with 5,000 bootstrap samples to estimate the direct and indirect effects, as well as the moderating effect in the association between disability and QoL among older adults. The significance of the mediation was determined when the 95% bias-corrected bootstrap confidence interval (CI) did not contain zero (Table 3).

**Table 2:** Factors associated with health-related quality of life among older adults.

Variables	Model 1			Model 2			Model 3			Model 4(sleep quality)			Model 5(depression)		
	OR	95% CI		OR	95% CI		OR	95% CI		B	95% CI		B	95% CI	
		Lower	Upper		Lower	Upper		Lower	Upper		Lower	Upper		Lower	Upper
Disability (*X*)	1.928^***^	1.707	2.177	1.846^***^	1.636	2.084	1.816^***^	1.606	2.053	0.277^***^	0.158	0.397	0.406^***^	0.305	0.507
Sleep quality (*M*1)				1.136^***^	1.114	1.159	1.031^*^	1.005	1.058						
Depression (*M*2)							1.198^***^	1.163	1.235						
Living arrangement (*W*)										−3.379^**^	−5.624	−1.135	−4.026^***^	−5.919	−2.133
Disability*Living arrangement (*XW*)										0.609^**^	0.255	0.963	0.739^***^	0.440	1.037
Sex	1.278	0.970	1.683	1.058	0.796	1.406	1.080	0.803	1.454	1.237^***^	0.827	1.646	0.590^**^	0.246	0.0934
Age	1.010	0.994	1.025	1.010	0.994	1.026	1.017^*^	1.000	1.033	−0.004	−0.028	0.021	−0.035^**^	−0.056	−0.014
Degree of education	0.845^***^	0.761	0.939	0.856^***^	0.768	0.954	0.878^*^	0.786	0.982	−0.189^*^	−0.345	−0.034	−0.234^***^	−0.364	−0.104
Health insurance	0.911	0.763	1.086	0.935	0.711	1.228	0.937	0.707	1.242	0.049	−0.329	0.426	0.008	−0.308	0.325
Household income	0.844^***^	0.772	0.922	0.865^***^	0.790	0.948	0.890^*^	0.810	0.979	−0.201^**^	−0.343	−0.060	−0.222^***^	−0.341	−0.103
Smoking status	0.911	0.763	1.086	0.898	0.749	1.077	0.918	0.761	1.108	0.046	−0.205	0.297	−0.076	−0.287	0.135
Alcohol consumption	1.059	0.970	1.155	1.045	0.955	1.143	1.031	0.939	1.131	0.065	−0.058	0.188	0.097	−0.006	0.201
Exercise	0.790^***^	0.742	0.841	0.794^***^	0.744	0.847	0.831^***^	0.777	0.889	−0.114^*^	−0.212	−0.015	−0.302^***^	−0.385	−0.220
Chronic disease	0.249^***^	0.189	0.329	0.318^***^	0.239	0.422	0.341^***^	0.255	0.456	−1.987^***^	−2.302	−1.673	−1.517^***^	−1.781	−1.253

Models 1–3 were estimated using binary logistic regression and are presented as odds ratios (ORs) with 95% confidence intervals (CIs). Models 4 and 5 were estimated using linear regression and are presented as unstandardized regression coefficients (B) with 95% CIs. ^***^p < 0.001, ^**^p < 0.01, ^*^p < 0.05; CI, confidence interval; *X*, independent variable; *M*, mediating variable; *W*, Moderating variable; *XW*, interaction term between *X* and *M*.

**Table 3:** Mediation of disability and quality of life by sleep quality and depression.

Paths	B	SE	*t/Z*	*p*	95% CI
1. Disability → sleep quality	0.424	0.059	7.152	<0.001	0.307 ~ 0.540
Sleep quality → quality of life	0.041	0.013	3.302	<0.001	0.017 ~ 0.066
2. Disability → depression	0.332	0.039	8.562	<0.001	0.256 ~ 0.407
Depression → quality of life	0.201	0.015	13.286	<0.001	0.171 ~ 0.231
3. Disability → sleep → depression	0.424	0.059	7.152	<0.001	0.307 ~ 0.540
Depression → quality of life	0.201	0.015	13.286	<0.001	0.171 ~ 0.231
4. Disability → quality of life	0.636	0.064	9.996	<0.001	0.512 ~ 0.761

CI, confidence interval; *B*, unstandardized regression coefficient; SE, standard error. *t*-values are reported for linear regression models, and *Z*-values for logistic regression models. Paths 1, 2, and 3 represent indirect paths (including the serial mediation path); Path 4 represents the direct path of disability on quality of life.

## 3 Results

### 3.1 Sample characteristics and univariate analysis related to QoL

A total of 3,855 older adults aged 60 years and above were included in this survey. Participant characteristics, along with the results of the univariate analysis of QoL, are presented in Table 1. Among the participants, 2,293 (59.5%) were female, indicating a higher proportion of female participants than male participants. The mean age of the respondents was 70.06 years, with a standard deviation of 6.22. The prevalence of disability among older adults was 4.8%. The mean disability score was 6.217 ± 1.217. In addition, 3,121 (81.0%) respondents were classified as having good QoL. The results indicated that the mean PHQ-9 score among older adults was 5.680 ± 4.503. Among those with good QoL, the mean score was 5.029 ± 4.089. In contrast, older adults with poor QoL had a higher mean score of 8.428 ± 5.098. The mean PHQ-9 score among older adults for depression was 3.046 ± 3.834. Among participants with good QoL, the mean score was 2.295 ± 2.887.

Table 1 also presents the results of the univariate analysis of factors associated with QoL. This analysis examined the relationships between QoL and a range of variables. These variables included sociodemographic characteristics, economic status-related factors, lifestyle-related factors, health-related factors, disability, sleep quality, and depression. The analysis indicated that several variables were significantly associated with QoL. These included sex (*χ*^2^ = 19.916, *p* < 0.001), age (*t* = −5.900, *p* < 0.001), education level (*χ*^2^ = 63.637, *p* < 0.001), health insurance (*χ*^2^ = 23.874, *p* < 0.001), annual household income (*χ*^2^ = 48.189, *p* < 0.001), smoking status (*χ*^2^ = 8.805, *p* < 0.001), alcohol consumption (*χ*^2^ = 22.842, *p* < 0.001), exercise (*χ*^2^ = 84.489, *p* < 0.001), chronic disease (*χ*^2^ = 139.066, *p* < 0.001), disability (*t* = −20.077, *p* < 0.001), sleep quality (*t* = −19.191, *p* < 0.001), and depression (*t* = −27.418, *p* < 0.001).

### 3.2 Factors associated with QoL and the moderating effect of living arrangement

#### 3.2.1 Correlates of QoL

Binomial logistic regression and linear regression analysis were conducted to examine the association between disability and QoL. Details were presented in Table 2. In Model 1, after controlling for covariates, disability was a strong and significant predictor of poor QoL [OR = 1.928, 95% CI (1.707, 2.177), *p* < 0.001]. This association remained significant after sequentially adding sleep quality (Model 2: OR = 1.846, *p* < 0.001) and depression (Model 3: OR = 1.816, *p* < 0.001). This stepwise attenuation of the effect size of disability, alongside significant associations of sleep quality and depression with QoL, provides preliminary support for the potential mediating roles of these two variables, which are formally tested in subsequent mediation analyses.

#### 3.2.2 Moderating effect of living arrangement

The moderating effect of living arrangement was tested using linear regression models (Models 4 and 5 in Table 2), with all analyses adjusted for covariates. The results showed that disability was positively associated with poorer sleep quality (B = 0.277, 95% CI: 0.158–0.397, *p* < 0.001) and higher depressive symptoms (B = 0.406, 95% CI: 0.305–0.507, *p* < 0.001). Most importantly, the interaction term between disability and living arrangement was significantly associated with both sleep quality (B = 0.609, 95% CI: 0.255–0.963, *p* < 0.01) and depression (B = 0.739, 95% CI: 0.440–1.037, *p* < 0.001). These findings indicate that living arrangement moderates the associations of disability with sleep quality and depressive symptoms among older adults.

### 3.3 Mediating roles of sleep quality and depression in the association between disability and QoL

Mediation analysis was conducted using the SPSS PROCESS macro (Version 4.0) to examine the parallel and serial mediating effects of sleep quality and depressive symptoms, and the core results were summarized in Tables 3, 4. Table 3 reports segmented unstandardized regression coefficients for each constituent path of the mediation model, after adjustment for all predefined covariates. First, disability exerted a significantly positive predictive effect on sleep quality (B = 0.424, *p* < 0.001, 95% CI: 0.307, 0.540), and sleep quality was significantly associated with reduced quality of life (B = 0.041, *p* < 0.001, 95% CI: 0.017, 0.066). Second, disability was positively associated with depressive symptoms (B = 0.332, *p* < 0.001, 95% CI: 0.256 ~ 0.407), and higher depression levels were significantly associated with poorer QoL (B = 0.201, *p* < 0.001, 95% CI: 0.171, 0.231). Meanwhile, disability positively predicted depression via sleep quality in the serial pathway (B = 0.424, *p* < 0.001, 95% CI: 0.307, 0.540). After accounting for the two mediators, the remaining direct effect of disability on QoL remained statistically significant (B = 0.636, *p* < 0.001, 95% CI: 0.512, 0.761).

**Table 4:** Direct and indirect effects of disability on quality of life.

Effect	Effect value	SE	95% CI	Proportion of total effect (%)
Total effect	0.767	__	__	100.00
Direct effect	0.637	0.064	(0.512 ~ 0.761)	83.00
Total indirect effect	0.130	0.021	(0.094 ~ 0.175)	17.00
Indirect effect 1: disability → sleep quality → QoL	0.017	0.006	(0.007 ~ 0.031)	2.28
Indirect effect 2: disability → depression → QoL	0.067	0.015	(0.040 ~ 0.098)	8.69
Indirect effect 3: disability → sleep quality → depression → QoL	0.046	0.010	(0.030 ~ 0.068)	6.03

CI, confidence interval; SE, standard error.

Table 4 further decomposes the total, direct, and three distinct indirect effects derived from the multiplication of the segmented path coefficients in Table 3, with the proportional contribution of each pathway against the total effect also reported where appropriate. The total effect of disability on QoL was 0.767. The direct effect of disability on QoL was B = 0.637 (95% CI: 0.512–0.761), accounting for 83.00% of the total effect. The aggregated total indirect effect across three mediation pathways was B = 0.130 (95% CI: 0.094–0.175), accounting for 17.00% of the total effect. Specifically, three statistically significant indirect pathways were confirmed. The indirect effect through sleep quality alone was significant (B = 0.017, 95% CI: 0.007–0.031), accounting for 2.28% of the total effect. The indirect effect through depression alone was significant (B = 0.067, 95% CI: 0.040–0.098), accounting for 8.69% of the total effect. Furthermore, the serial indirect effect through sleep quality and depression was significant (B = 0.046, 95% CI: 0.030–0.068), accounting for 6.03% of the total effect. Collectively, these results support the presence of partial parallel and serial mediation by sleep quality and depression in the association between disability and QoL.

**Figure 3:**
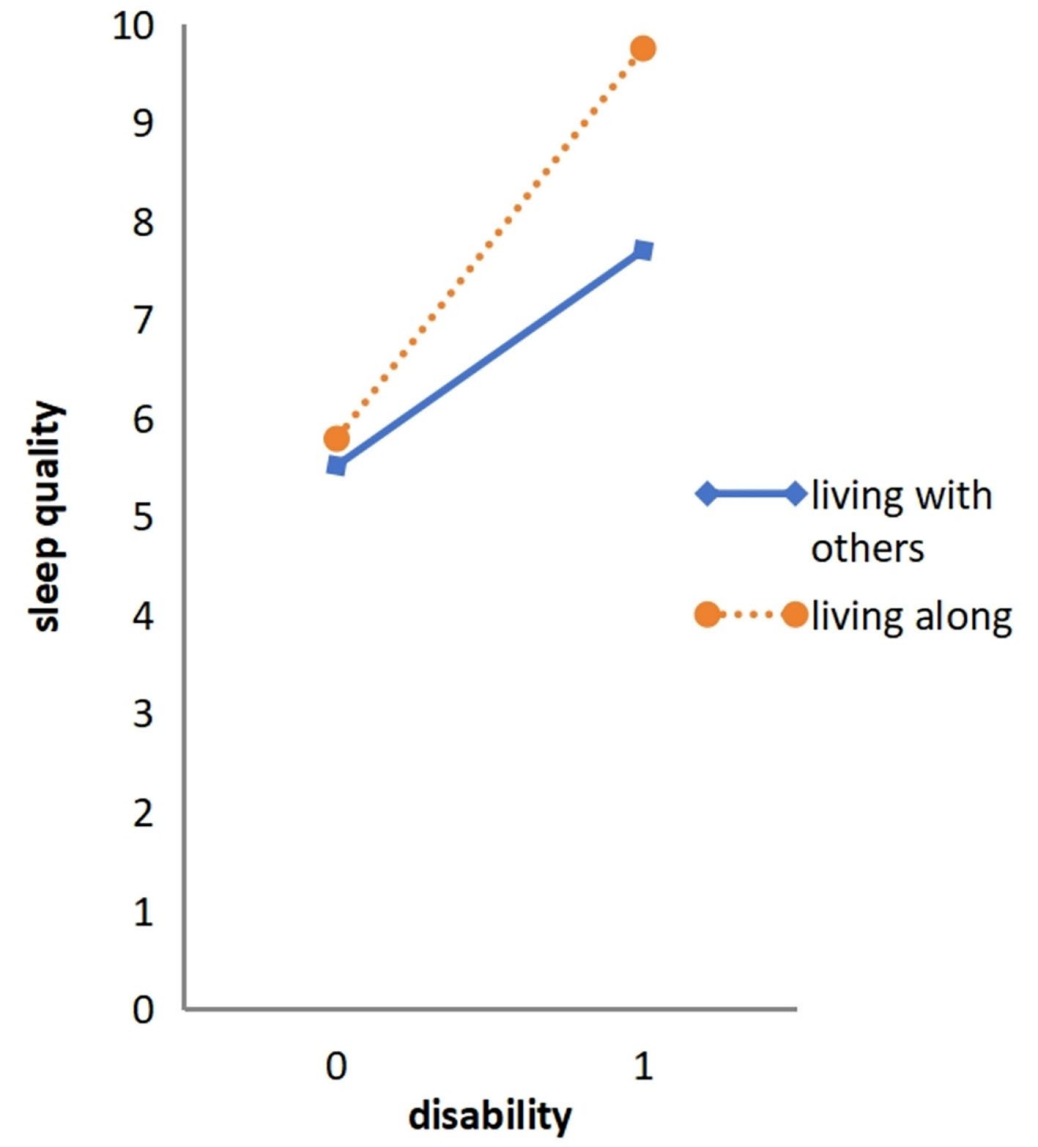
The moderating effect of living arrangements on disability and sleep quality.

**Figure 4:**
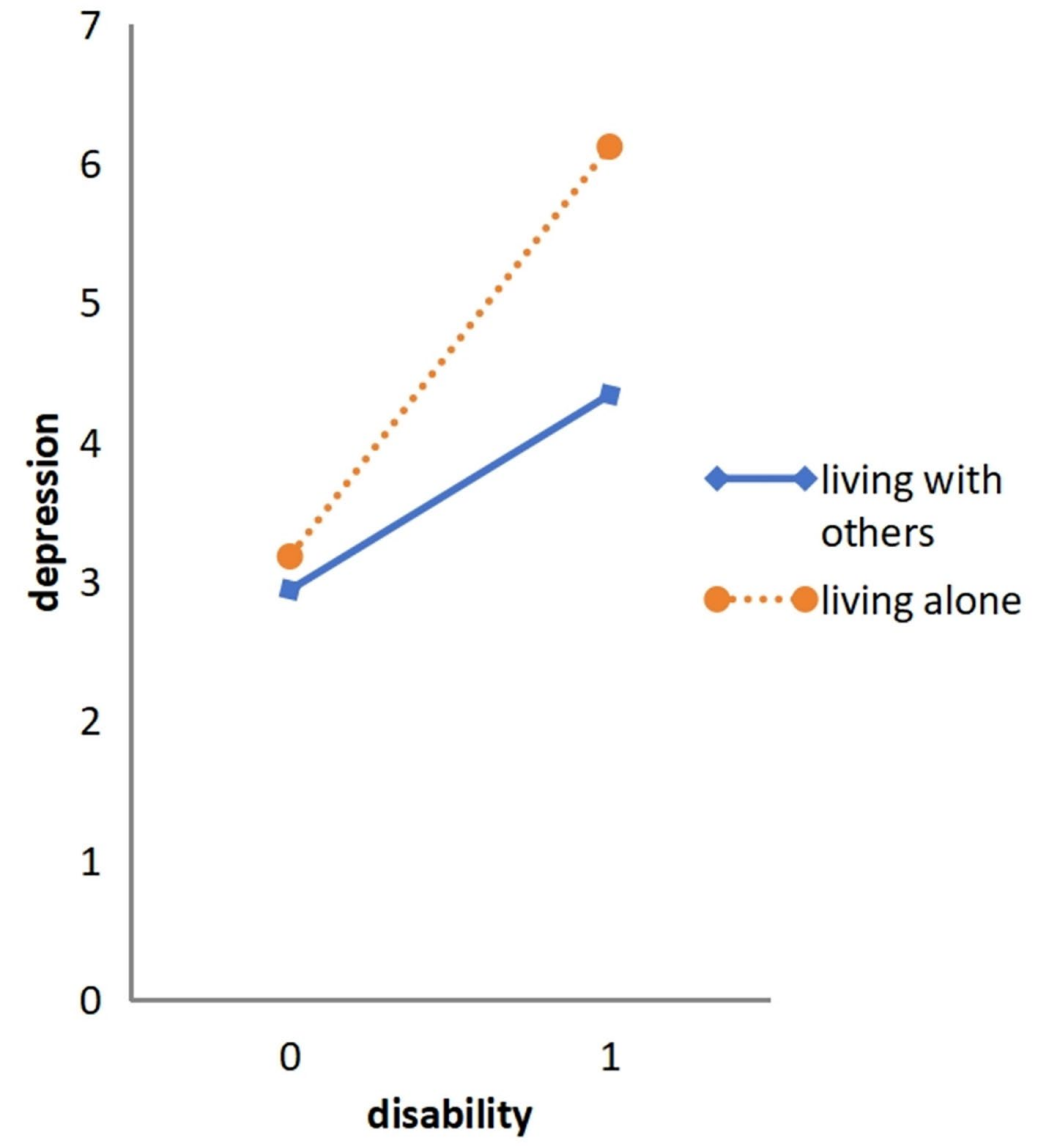
The moderating effect of living arrangements on disability and depression.

To further examine the interaction effect between disability and living arrangements, living arrangements were catagorized into living alone and not living alone. Simple slope analysis (see Figures 3, 4) indicated that the positive associations between disability and poor sleep quality, as well as between disability and depression, were stronger among older adults living alone than among those who were not living alone.

## 4 Discussion

This study examined the association between disability and QoL among older adults within a biopsychosocial framework. In this study, the prevalence of disability was 4.8%, which was lower than that reported in previous national and international studies, possibly due to differences in measurement criteria and population characteristics (48–50). Disability was a significant risk factor negatively associated with QoL among older adults, which is consistent with previous research (51). This relationship has also been observed across different populations (52). Sleep quality and depression partially mediated this relationship, forming a serial mediation pathway. In addition, living arrangements moderated the effects of disability on both sleep quality and depression, with stronger adverse effects observed among older adults living alone.

The present findings highlight the importance of biological and psychological pathways in explaining how disability is linked to QoL. Our results confirmed that disability was negatively associated with sleep quality in older adults. From a biological perspective, disability is often accompanied by chronic diseases, physical pain, and functional limitations, which may impair sleep quality (53, 54). These challenges are particularly evident among severely disabled individuals, who experience difficulties with basic nighttime activities such as repositioning and toileting (55). Beyond normal age-related sleep changes, disability further exacerbates sleep disturbances through persistent discomfort and restricted mobility (56). Poor sleep, in turn, has been associated with cognitive decline, increased risk of chronic diseases, and reduced overall health status, ultimately contributing to lower QoL (20).

This study also found that disability significantly increased depressive symptoms among older adults. From a psychological perspective, reduced functional capacity may limit social participation and lead to feelings of helplessness and uselessness, thereby increasing depressive symptoms and reducing QoL (57). Furthermore, our sequential mediation results also verified that poor sleep quality further elevated depressive symptoms, which in turn decreased QoL. Sleep disturbances have been identified as both a core symptom and a predictive factor of depression, with epidemiological studies indicating that insomnia significantly increases the risk of subsequent depression (58). Continuous poor-quality sleep may impair emotional regulation and increase psychological vulnerability, suggesting a close interaction between sleep and depression. Moreover, disability may also trigger psychosocial stress pathways, including loneliness, reduced social engagement, and increased emotional burden, which further exacerbate both sleep disturbances and depressive symptoms (24, 59). Additionally, the loss of labor capacity may lead to reduced income, and the increased disease-related financial burden may intensify anxiety and depression, thereby contributing to impaired QoL (60, 61). Taken together, these findings provide strong support for a serial mediation pathway in which disability is linked to sleep quality, which subsequently exacerbates depressive symptoms, ultimately leading to lower QoL within a biopsychosocial framework.

This study identified a moderating effect of living arrangements in the mediation model, highlighting the critical role of social factors. Living with family members significantly mitigated the association of disability with both sleep disturbances and depression among older adults, which is consistent with the social support stress-buffering model (26). This buffering effect may be attributed to stronger social support networks among co-residing older adults, including both emotional and instrumental support (62). For instance, assistance with daily and nighttime activities, such as toileting and repositioning, may reduce sleep disruptions caused by physical discomfort. Emotional support from family members may also alleviate psychological distress and reduce depressive symptoms. In contrast, older adults living alone may experience reduced access to social and material support and increased concerns about emergencies, which may be related to sustained psychological hypervigilance and poorer sleep quality (63, 64). According to the stress susceptibility framework, living alone may exacerbate vulnerability to disability-related stressors and amplify their negative effects on mental health outcomes such as insomnia and depression (27, 63). These findings emphasize the importance of social context in shaping health outcomes among older adults.

Altogether, disability was strongly associated with QoL among older adults. Sleep quality and depression mediated the relationship between disability and QoL, while living arrangements moderated the first half of the mediation pathway. These findings provide empirical support for the biopsychosocial model, indicating that the association of disability with QoL operates through interconnected biological, psychological, and social mechanisms. From a public health perspective, interventions aimed at improving QoL among older adults with disabilities should go beyond physical rehabilitation. Integrated care strategies addressing sleep quality, mental health, and social support are warranted. Initial efforts should include screening for sleep disorders and depression, establishing risk profiles, and ensuring timely intervention. Furthermore, community-based services, regular follow-up, and telecare programs may be particularly beneficial for disabled older adults living alone. In addition, strengthening financial support, promoting sleep hygiene, and improving home environments may help create a supportive context for healthy aging.

Several strengths and limitations should be acknowledged. This study benefits from a relatively large sample size and an integrated analytical framework that simultaneously examines mediation and moderation mechanisms. However, several limitations should be considered. First, the cross-sectional design of this study precludes causal inference and limits the ability to establish temporal ordering among disability, sleep quality, depression, and quality of life. As such, the observed associations cannot be interpreted as causal relationships. Future studies should adopt longitudinal or interventional designs to further clarify the causal pathways underlying these relationships. Second, the data were obtained from a single city, which may limit the generalizability of the findings. Future research should expand the sampling scope to improve external validity. Third, the use of self-reported measures may introduce recall bias. Future studies could incorporate objective measurements, such as actigraphy for sleep assessment, to enhance measurement accuracy.

## 5 Conclusion

In conclusion, this study elucidates a complex biopsychosocial pathway through which disability is associated with QoL among older adults. The main findings are as follows: (1) Disability not only has a direct negative effect on QoL but also exerts indirect effects through a sequential mediating pathway involving poor sleep quality and depression; and (2) Living arrangements moderate the first stage of this chain mediation, and living alone exacerbates the adverse association of disability with sleep quality and depressive symptoms in the present study. These findings highlight the need for targeted public health strategies to improve QoL in older adults with disabilities, especially those living alone. A comprehensive, integrated approach is warranted: routine screening and early intervention for sleep problems and depression should be incorporated into routine care for older adults with disabilities. In addition, enhanced financial support, smart older adult-friendly home modifications, and expanded social support services are critical to buffer the negative psychophysiological associations of disability. Together, these efforts can effectively promote healthy and active aging in this vulnerable population.

## Data Availability

The raw data supporting the conclusions of this article will be made available by the authors upon reasonable request.

## References

[1] National Bureau of Statistics. Bulletin of the Seventh National Population Census (2021). Available online at: https://proapi.jingjiribao.cn/detail.html?id=339961 (Accessed June 20, 2026).

[2] National Health Commission of the People's Republic of China. China's elderly population to hit 400 mln by 2035: expert (2017). Available online at: https://en.nhc.gov.cn/2017-10/29/c_72599.htm. (Accessed June 20, 2026).

[3] National Health Commission of the People's Republic of China. Opinions of the Central Committee of the Communist Party of China and the State council on strengthening the work of aging in the new era (2021). Available online at: https://www.nhc.gov.cn/wjw/mtbd/202111/478268ea4939419b92bfbbf5252ac59f.shtml. (Accessed June 20, 2026).

[4] National Health Commission of the People's Republic of China. "The national long-term plan for actively Responding to population aging" elevates the issue of aging to a national strategy (2019). Available online at: https://www.gov.cn/zhengce/2019-1/23/content_5454778.htm. (Accessed June 20, 2026).

[5] Genther DJ Frick KD Chen D Betz J Lin FR. Association of hearing loss with hospitalization and burden of disease in older adults. JAMA. (2013) 309:2322–4. doi: 10.1001/jama.2013.5912, 23757078 PMC3875309

[6] Beltz S Gloystein S Litschko T Laag S van den Berg N. Multivariate analysis of independent determinants of ADL/IADL and quality of life in the elderly. BMC Geriatr. (2022) 22:894. doi: 10.1186/s12877-022-03621-3, 36418975 PMC9682836

[7] World Health Organization. World report on ageing and health (2015). Available online at: https://www.who.int/publications/i/item/9789241565042. (Accessed June 20, 2026).

[8] World Health Organization. Active ageing: a policy framework (2002). Available online at: https://iris.who.int/handle/10665/67215. (Accessed June 20, 2026).

[9] Katz S Ford AB Moskowitz RW Jackson BA Jaffe MW. Studies of illness in the aged: the index of ADL: a standardized measure of biological and psychosocial function. JAMA. (1963) 185:914–9. doi: 10.1001/jama.1963.03060120024016 14044222

[10] Baernholdt M Hinton I Yan G Rose K Mattos M. Factors associated with quality of life in older adults in the United States. Qual Life Res. (2012) 21:527–34. doi: 10.1007/s11136-011-9954-z, 21706127 PMC3593634

[11] Thangiah G Said MA Majid HA Reidpath D Su TT. Income inequality in quality of life among rural communities in Malaysia: a case for immediate policy consideration. Int J Environ Res Public Health. (2020) 17:8731. doi: 10.3390/ijerph17238731, 33255397 PMC7727827

[12] Hou B Li Y Wang H. Internet use and health status among older adults: the mediating role of social participation. Front Public Health. (2022) 10:1072398. doi: 10.3389/fpubh.2022.1072398, 36504989 PMC9732719

[13] Oh E Moon S Hong GS. Longitudinal trends and predictors of limitations in activities of daily living in community-dwelling older adults: evidence from the KLoSA study. Front Public Health. (2024) 12:1485732. doi: 10.3389/fpubh.2024.1485732, 39735760 PMC11673221

[14] Havelka M Lucanin JD Lucanin D. A biopsychosocial model for the management of patients with sickle-cell disease transitioning to adult medical care. Coll Antropol. (2009) 32:293–305. doi: 10.1007/s12325-015-0197-1, 25832469 PMC4415939

[15] Sokas C Herrera-Escobar JP Klepp T Stanek E Kaafarani H Salim A . Impact of chronic illness on functional outcomes and quality of life among injured older adults. Injury. (2021) 52:2638–44. doi: 10.1016/j.injury.2021.03.052, 33823987

[16] Motl RW McAuley E. Physical activity, disability, and quality of life in older adults. Phys Med Rehabil Clin N Am. (2010) 21:299–308. doi: 10.1016/j.pmr.2009.12.006, 20494278

[17] Tajika T Kuboi T Endo F Shinagawa S Kobayashi H Hashimoto S . Association between upper extremity dysfunction and sleep disturbance in an elderly general population. SAGE Open Med. (2020) 8:2050312120901584. doi: 10.1177/2050312120901584, 32030126 PMC6977088

[18] Cao Y Yang Z Yu Y Huang X. Physical activity, sleep quality and life satisfaction in adolescents: a cross-sectional survey study. Front Public Health. (2022) 10:1010194. doi: 10.3389/fpubh.2022.1010194, 36605236 PMC9807806

[19] Cai D Zeng Y Chen M Zhong Y Quan Y Ye M . Association between sleep duration and disability in activities of daily living among Chinese older adults: a nationwide observational study. Front Public Health. (2025) 13:1580101. doi: 10.3389/fpubh.2025.1580101, 40469604 PMC12133472

[20] Wu Y Chen Z Cheng Z Yu Z Qin K Jiang C . Effects of chronic diseases on health related quality of life is mediated by sleep difficulty in middle aged and older adults. Sci Rep. (2025) 15:2987. doi: 10.1038/s41598-025-86420-1, 39849013 PMC11758026

[21] Jiang J Tang Z Futatsuka M. The impact of ADL disability on depression symptoms in a community of Beijing elderly, China. Environ Health Prev Med. (2002) 7:199–204. doi: 10.1007/BF02898005, 21432278 PMC2723587

[22] Yan Y Du Y Li X Ping W Chang Y. Physical function, ADL, and depressive symptoms in Chinese elderly: evidence from the CHARLS. Front Public Health. (2023) 11:1017689. doi: 10.3389/fpubh.2023.1017689, 36923048 PMC10010774

[23] Papageorgiou A Bakola M Kitsou K Mousafeiris V Mavridou K Kallianezos P . The association between depression and quality of life in the elderly. Eur J Pub Health. (2022) 32:ckac131.125. doi: 10.1093/eurpub/ckac131.125

[24] Franzen PL Buysse DJ. Sleep disturbances and depression: risk relationships for subsequent depression and therapeutic implications. Dialogues Clin Neurosci. (2008) 10:473–81. doi: 10.31887/DCNS.2008.10.4/plfranzen, 19170404 PMC3108260

[25] Wu Y Zhao L Man X Jiang Y Zhang L. The impact of disability status on depression in the aged with the moderating effect of community support. BMC Psychol. (2025) 13:1067. doi: 10.1186/s40359-025-03289-5, 41023753 PMC12482041

[26] Cohen S Wills TA. Stress, social support, and the buffering hypothesis. Psychol Bull. (1985) 98:310–57. doi: 10.1037/0033-2909.98.2.310 3901065

[27] Saito T Murata C Aida J Kondo K. Cohort study on living arrangements of older men and women and risk for basic activities of daily living disability: findings from the AGES project. BMC Geriatr. (2017) 17:m183. doi: 10.1186/s12877-017-0580-7, 28814289 PMC5559833

[28] Choi WS Kang SW Choi SB. The dark side of mobile work during non-work hours: moderated mediation model of presenteeism through conservation of resources lens. Front Public Health. (2024) 12:1186327. doi: 10.3389/fpubh.2024.1186327 38439760 PMC10909990

[29] Shang Z Liu Y Xue D Zheng Y Li Y Zhang B . The role of life satisfaction and living arrangements in the association between chronic disease and depression: a national cross-sectional survey. Front Psychol. (2023) 14:1266059. doi: 10.3389/fpsyg.2023.1266059, 37965656 PMC10641446

[30] Taian Municipal Bureau of Statistics. Bulletin of the Seventh National Population Census in Taian City (2021). Available online at: https://tjj.taian.gov.cn/art/2021/10/28/art_46885_10287034.html. (Accessed June 20, 2026).

[31] Taian Municipal People's Government. Comprehensive solution for "The Elderly and Children" in Taian City (2022). Available online at: https://www.taian.gov.cn/art/2022/12/27/art_317829_3105.html. (Accessed June 20, 2026).

[32] Zhu J Xu L Sun L Qin D. Negative life events, sleep quality, and depression among older adults in Shandong Province, China: a conditional process analysis based on economic income. Geriatr Gerontol Int. (2024) 24:751–7. doi: 10.1111/ggi.14914, 39089878 PMC11503548

[33] You R Liu J Yang Z Pan C Ma Q Luo N. Comparing the performance of the EQ-5D-3 L and the EQ-5D-5 L in an elderly Chinese population. Health Qual Life Outcomes. (2020) 18:97. doi: 10.1186/s12955-020-01324-0, 32272976 PMC7147057

[34] Yang Z Busschbach J Liu G Luo N. EQ-5D-5L norms for the urban Chinese population in China. Health Qual Life Outcomes. (2018) 16:210. doi: 10.1186/s12955-018-1036-2, 30409137 PMC6225616

[35] Luo N Liu G Li M Guan H Jin X Rand-Hendriksen K. Estimating an EQ-5D-5L value set for China. Value Health. (2017) 20:662–9. doi: 10.1016/j.jval.2016.11.016, 28408009

[36] Su M Yao N Shang M Shen Y Qin T Wang J . Frailty and its association with health-related quality of life among older cancer patients: an evidence-based study from China. Health Qual Life Outcomes. (2022) 20:124. doi: 10.1186/s12955-022-02032-7, 35986354 PMC9389706

[37] Kim MJ Park S Jung YI Kim SH Oh IH. Exploring health-related quality of life and frailty in older adults based on the Korean frailty and aging cohort study. Qual Life Res. (2020) 29:2911–9. doi: 10.1007/s11136-020-02568-5, 32607792

[38] Self-maintenance P. Assessment of older people: self-maintaining and instrumental activities of daily living. Gerontologist. (1969) 9:179–86. doi: 10.1093/geront/9.3_Part_1.179 5349366

[39] Buysse DJ Reynolds CF 3rd Monk TH Berman SR Kupfer DJ. The Pittsburgh sleep quality index: a new instrument for psychiatric practice and research. Psychiatry Res. (1989) 28:193–213. doi: 10.1016/0165-1781(89)90047-4, 2748771

[40] Wang L Wu YX Lin YQ Wang L Zeng ZN Xie XL . Reliability and validity of the Pittsburgh sleep quality index among frontline COVID-19 health care workers using classical test theory and item response theory. J Clin Sleep Med. (2022) 18:541–51. doi: 10.5664/jcsm.9658, 34534069 PMC8805004

[41] Beswick E Quigley S Macdonald P Patrick S Colville S Chandran S . The patient health questionnaire (PHQ-9) as a tool to screen for depression in people with multiple sclerosis: a cross-sectional validation study. BMC Psychol. (2022) 10:281. doi: 10.1186/s40359-022-00949-8, 36443880 PMC9706934

[42] Sun Y Fu Z Bo Q Mao Z Ma X Wang C. The reliability and validity of PHQ-9 in patients with major depressive disorder in psychiatric hospital. BMC Psychiatry. (2020) 20:474. doi: 10.1186/s12888-020-02885-6, 32993604 PMC7525967

[43] Hu X Liu H Liu Q Yuan T Duan M Luo Y . Depressive symptoms and their influencing factors among older adults in China: a cross-sectional study. Front Public Health. (2024) 12:1423391. doi: 10.3389/fpubh.2024.1423391 39618958 PMC11605914

[44] Zheng Y Gao M Hou G Hou N Feng X Jannini TB . A prospectively validated nomogram for predicting the risk of PHQ-9 score ≥15 in patients with erectile dysfunction: a multi-center study. Front Public Health. (2022) 10:836898. doi: 10.3389/fpubh.2022.836898, 35784263 PMC9247334

[45] Jiang F Zhang J Qin W Ding G Xu L. Hearing impairment and loneliness in older adults in Shandong, China: the modifying effect of living arrangement. Aging Clin Exp Res. (2021) 33:1015–21. doi: 10.1007/s40520-020-01594-0, 32557333

[46] Yu D Shimura M Kawanishi M. Relationship between regular exercise and quality of life among middle-aged and older adults in Japan. Behav Sci. (2025) 15:978. doi: 10.3390/bs15070978, 40723762 PMC12292658

[47] Zhao Q Li X Chen H Wang L Wu N Ma J . Association between depression and pain, functional disability, disease activity and health-related quality of life in patients with systemic lupus erythematosus: a meta-analysis. BMJ Open. (2023) 13:e068683. doi: 10.1136/bmjopen-2022-068683 PMC10583069 37821132

[48] Zheng W Huang Z. Onset of ADL and IADL limitation among Chinese middle-aged and older adults. PLoS One. (2023) 18:e0287856. doi: 10.1371/journal.pone.0287856, 37459324 PMC10351716

[49] Germain CM Vasquez E Batsis JA McQuoid DR. Sex, race and age differences in muscle strength and limitations in community dwelling older adults: data from the Health and Retirement Survey (HRS). Arch Gerontol Geriatr. (2016) 65:98–103. doi: 10.1016/j.archger.2016.03.007, 27017414

[50] Yoshida D Ninomiya T Doi Y Hata J Fukuhara M Ikeda F . Prevalence and causes of functional disability in an elderly general population of Japanese: the Hisayama study. J Epidemiol. (2012) 22:222–9. doi: 10.2188/jea.je20110083, 22343328 PMC3798623

[51] Chatterji S Byles J Cutler D Seeman T Verdes E. Health, functioning, and disability in older adults—present status and future implications. Lancet. (2015) 385:563–75. doi: 10.1016/s0140-6736(14)61462-8, 25468158 PMC4882096

[52] Gobbens RJ Remmen R. The effects of sociodemographic factors on quality of life among people aged 50 years or older are not unequivocal: comparing SF-12, WHOQOL-BREF, and WHOQOL-OLD. Clin Interv Aging. (2019) 14:231–9. doi: 10.2147/CIA.S189560, 30787599 PMC6363394

[53] Zhang M Zhu W He X Liu Y Sun Q Ding H. Correlation between functional disability and quality of life among rural elderly in Anhui province, China: a cross-sectional study. BMC Public Health. (2022) 22:397. doi: 10.1186/s12889-021-12363-7 35216578 PMC8881859

[54] Shen QQ Hu LL Geng SJ Cui L. The association between physical activity and depression in emerging adults: the pathway of subjective exercise experience. Front Public Health. (2026) 13:1718409. doi: 10.3389/fpubh.2025.1718409, 41696699 PMC12898816

[55] Pollak CP Perlick D Linsner JP Wenston J Hsieh F. Sleep problems in the community elderly as predictors of death and nursing home placement. J Community Health. (1990) 15:123–35. doi: 10.1007/bf01321316, 2355110

[56] Gulia KK Kumar VM. Sleep disorders in the elderly: a growing challenge. Psychogeriatrics. (2018) 18:155–65. doi: 10.1111/psyg.12319, 29878472

[57] Telebuh M Havelka M Bertić Ž Čovčić GG Grubišić M Jakuš L . Functional mobility and depression negatively impact quality of life in older adults with stroke. NeuroRehabilitation. (2025) 55:448–58. doi: 10.1177/10538135241291373 42543860

[58] Nutt D Wilson S Paterson L. Sleep disorders as core symptoms of depression. Dialogues Clin Neurosci. (2008) 10:329–36. doi: 10.31887/DCNS.2008.10.3/dnutt 18979946 PMC3181883

[59] Liu D Lei G Qiu L Deng H Liu S Dang Y . Rumination and depression in Chinese adolescents with mood disorders: the mediating role of resilience. J Clin Psychiatry. (2023) 84:22m14682. doi: 10.4088/JCP.22m14682, 37498649

[60] Louie GH Ward MM. Socioeconomic and ethnic differences in disease burden and disparities in physical function in older adults. Am J Public Health. (2011) 101:1322–9. doi: 10.2105/ajph.2010.199455, 21164082 PMC3110229

[61] Scanlan JM Binkin N Michieletto F Lessig M Zuhr E Borson S. Cognitive impairment, chronic disease burden, and functional disability: a population study of older Italians. Am J Geriatr Psychiatry. (2007) 15:716–24. doi: 10.1097/JGP.0b013e3180487cd7 17567931

[62] Jin F Hu Y. Association between living arrangements, social support, and depression among middle-aged and older adults: a mediation analysis from the CHARLS survey. Front Psychol. (2025) 16:1492495. doi: 10.3389/fpsyg.2025.1492495, 39995427 PMC11847811

[63] Kobayashi E Fujiwara Y Fukaya T Nishi M Saito M Shinkai S. Social support availability and psychological well-being among the socially isolated elderly. Differences by living arrangement and gender. Jpn J Public Health. (2011) 58:446–56. doi: 10.1093/geronb/gbr104 21970078

[64] Bouaziz G Brulin D Pigot H Campo E. Detection of social isolation based on meal-taking activity and mobility of elderly people living alone. IRBM. (2023) 44:100770. doi: 10.1016/j.irbm.2023.100770

